# Supplementary material for: OpenMS WebApps: Building User-Friendly Solutions for MS Analysis
Source: J Proteome Res. 2025 Jan 30;24(2):940–8. doi: 10.1021/acs.jproteome.4c00872 (PMC11811998; doi:10.1021/acs.jproteome.4c00872)
Supplement: Supplementary file 1 — pr4c00872_si_001.pdf [file pr4c00872_si_001.pdf]

SUPPLEMENTARY INFORMATION

OpenMS WebApps: Building User-Friendly Solutions for MS Analysis

Tom David Müller †[1,2], Arslan Siraj †[1,2], Axel Walter[1,2], Jihyung Kim[1,2], Samuel Wein[1,2], Johannes von Kleist[1], Ayesha Feroz[1,2], Matteo Pilz[1,2], Kyowon Jeong[1,2], Justin Cyril Sing[3,4], Joshua Charkow[3,4], Hannes Luc Röst*[3,4], Timo Sachsenberg*[1,2]

† Equal Contribution

1. Applied Bioinformatics, Department of Computer Science, University of Tübingen, Tübingen, Germany
2. Institute for Bioinformatics and Medical Informatics, University of Tübingen, Tübingen, Germany
3. Donnelly Centre for Cellular and Biomolecular Research, University of Toronto, Toronto, Ontario M5S 3E1, Canada
4. Department of Molecular Genetics, University of Toronto, Toronto, Ontario M5G 1A8, Canada

TABLE OF CONTENTS:

Supplementary Item	Description	Page
Supplementary Text S1	A detailed description for each currently existing WebApp.	2
Supplementary Figure S1	Running third party tools with OpenMS WebApps – an example demonstrating the integration of MSConvert with the template web application	4

Supplementary Text S1. Current OpenMS WebApps

FLASHViewer

FLASHViewer is a comprehensive web application designed for visualizing and analyzing results from the FLASH* tool suite in top-down proteomics. It provides highly configurable, modular visualization components, enabling users to customize layouts and view multiple experiments simultaneously. Users can visualize various aspects, including raw and deconvolved spectrum plots, MS1 heatmaps, mass tables, 3D signal to noise plots, and dynamic sequence plots annotated with fragment ions. The application offers deep interactivity between components, such as clicking a row in a table to update related visualizations such as spectrum plots.

NuXL-app

The NuXL-app is a web application version of the NuXL search engine, a specialized tool developed for the analysis of protein nucleic acid cross-linking mass spectrometry data. It allows for reliable, FDR-controlled assignment of protein–nucleic acid crosslinking sites from samples treated with UV light or chemical crosslinkers and offers user-friendly matched spectra visualization including ion annotations.

UmetaFlow

This OpenMS WebApp offers the powerful UmetaFlow[1] pipeline for untargeted metabolomics in an accessible user interface. Raw data pre-processing converts raw data to a feature quantification table by feature detection, alignment, grouping, adduct annotation and optional re-quantification of missing values. Features can be annotated by in-house libraries based on MS1 m/z and retention time matching as well as MS2 fragment spectrum similarity. Features can be annotated with sum formula, compound name and chemical class by SIRIUS[2,3]. For further investigation input files for GNPS feature-based molecular networking[4] and ion identity molecular networking[5] can be exported. Besides the untargeted pipeline, this app offers some additional features, such as an interface to explore raw data and metabolite identification and quantification via extracted ion chromatograms based on exact m/z values generated conveniently by an included m/z calculator. For downstream processing statistical analysis can be performed within the app or in the popular FBmn STATS GUIde[6].

NASEWEIS

The NucleicAcidSearchEngine (also known as NASE) is a tool to do library searching and modification detection and localization on oligonucleotide mass spectrometry data[7]. It offers a wide variety of options to customize searching, which is ideal for experienced users but carries with it a learning curve. The NucleicAcidSearchEngine Web Execution

In Streamlit (aka NASEWEIS), attempts to fill this gap by being a web-service version requiring no installation, geared for simple analysis of small experiments, and specifically only exposing the most important parameters to the user. NASEWEIS allows users to upload data from an MS experiment, a fasta file for (potentially modified) sequences to search, and set parameters regarding the resolution of the instrument. It then identifies candidates for Oligonucleotide spectral matches, and does FDR to return a table of Oligonucleotide hits, as well as an optional idXML output file for viewing the data with OpenMS' TOPPView.

SagePTMScanner

Sage is a proteomics search engine that provides a variety of features such as retention time prediction, FDR-control, chimera searching and open searches among others[8]. SagePTMScanner provides an integration of Sage in OpenMS WebApps and aims to remove several hurdles towards using this software: it provides reasonable presets for searches, includes annotation and PTM discovery and produces immediate graphical output.

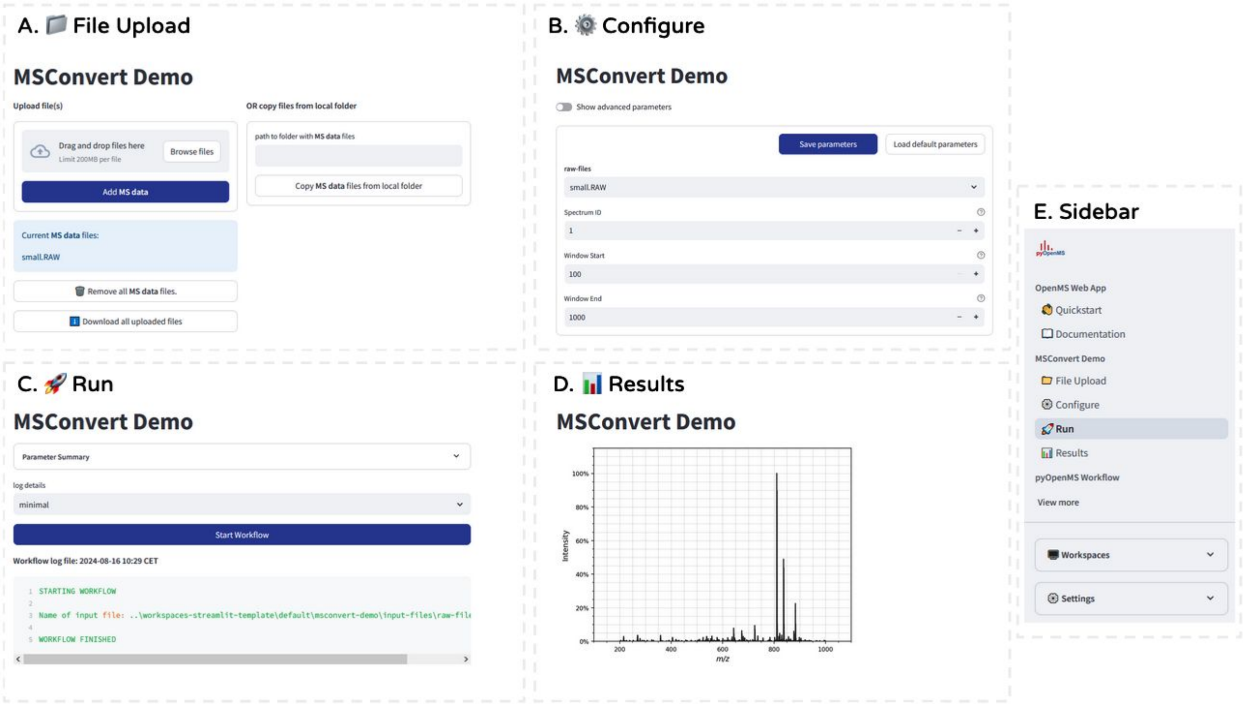

Supplementary Figure S1. Running third party tools with OpenMS WebApps: Third party tools can be used seamlessly with the WebApps framework. In this example MSConvert was integrated within the template. This mock workflow allows users to convert raw files to the mzML file format, select a single spectrum and m/z-range, and view the result as a graph. **A. File Upload:** Users can upload raw files. **B. Configure:** Users can select a single uploaded raw file, the spectrum ID and the m/z range. **C. Run:** MSConvert with the set parameters is run. **D. Results:** A simple plot of the resulting MSConvert output. **E. Sidebar:** The workflow can be navigated intuitively using the app's sidebar.

References

[1] E. E. Kontou *et al.*, “UmetaFlow: an untargeted metabolomics workflow for high-throughput data processing and analysis,” *J. Cheminformatics*, vol. 15, no. 1, p. 52, May 2023, doi: 10.1186/s13321-023-00724-w.Accessed: Sep. 05, 2024

[2] K. Dührkop, H. Shen, M. Meusel, J. Rousu, and S. Böcker, “Searching molecular structure databases with tandem mass spectra using CSI:FingerID,” *Proc. Natl. Acad. Sci.*, vol. 112, no. 41, pp. 12580–12585, Oct. 2015, doi: 10.1073/pnas.1509788112.Accessed: Sep. 18, 2024

[3] K. Dührkop *et al.*, “SIRIUS 4: a rapid tool for turning tandem mass spectra into metabolite structure information,” *Nat. Methods*, vol. 16, no. 4, pp. 299–302, Apr. 2019, doi: 10.1038/s41592-019-0344-8.Accessed: Sep. 18, 2024

[4] L.-F. Nothias *et al.*, “Feature-based molecular networking in the GNPS analysis environment,” *Nat. Methods*, vol. 17, no. 9, pp. 905–908, Sep. 2020, doi: 10.1038/s41592-020-0933-6.Accessed: Sep. 18, 2024

[5] R. Schmid *et al.*, “Ion identity molecular networking for mass spectrometry-based metabolomics in the GNPS environment,” *Nat. Commun.*, vol. 12, no. 1, p. 3832, Jun. 2021, doi: 10.1038/s41467-021-23953-9.Accessed: Sep. 18, 2024

[6] A. K. Pakkir Shah *et al.*, “Statistical analysis of feature-based molecular networking results from non-targeted metabolomics data,” *Nat. Protoc.*, pp. 92–162, Sep. 2024, doi: 10.1038/s41596-024-01046-3.Accessed: Sep. 24, 2024

[7] S. Wein *et al.*, “A computational platform for high-throughput analysis of RNA sequences and modifications by mass spectrometry,” *Nat. Commun.*, vol. 11, no. 1, p. 926, Feb. 2020, doi: 10.1038/s41467-020-14665-7.Accessed: Sep. 05, 2024

[8] M. R. Lazear, “Sage: An Open-Source Tool for Fast Proteomics Searching and Quantification at Scale,” *J. Proteome Res.*, vol. 22, no. 11, pp. 3652–3659, Nov. 2023, doi: 10.1021/acs.jproteome.3c00486.Accessed: Sep. 18, 2024
